# Marine subsidies produce cactus forests on desert islands

**DOI:** 10.1038/s41598-022-21133-3

**Published:** 2022-10-12

**Authors:** Benjamin T. Wilder, Amanda T. Becker, David L. Dettman

**Affiliations:** 1 Next Generation Sonoran Desert Researchers (N-Gen), Tucson, USA; 2 College of Education, University of Arizona, 1430 E 2nd St, Tucson, AZ 85721 USA; 3 Department of Geosciences, University of Arizona, 1040 E. 4th St, Tucson, AZ 85721 USA; 4 Estuary Research Center, Shimane University, 1060 Nishikawatsu-cho, Matsue-shi, Shimane, 690-8504 Japan

**Keywords:** Ecological networks, Plant ecology, Biogeography, Marine biology

## Abstract

In island systems, nitrogen-rich seabird guano is a marine subsidy that can shape terrestrial plant communities. In zones of nutrient upwelling such as the Gulf of California, copious seabird guano is commonplace on bird islands. Several bird islands host regionally unique cactus forests, especially of the large columnar cactus, cardón (*Pachycereus pringlei*). We show that a chain of interactions across the land-sea interface yields an allochthonous input of nitrogen in the form of seabird guano, fueling the production of some of the densest cactus populations in the world. Fish, seabird, guano, soil, and cactus samples were taken from the representative seabird island of San Pedro Mártir for nitrogen stable isotope ratio measurements, which were compared to soil and cactus samples from other seabird and non-seabird Gulf islands and terrestrial ecosystems throughout the range of the cardón. Isla San Pedro Mártir δ^15^N values are distinctively high, ranging from fish + 17.7, seabird + 19.7, guano + 14.8, soil + 34.3 and cactus + 30.3 compared to average values across non-bird sites of + 13.0 (N = 213, S.D. = 3.7) for soil and + 9.8 (N = 212, S.D. = 3.4) for cactus. These δ^15^N values are among the highest ever reported for plants. Seabird island soil and cactus δ^15^N values were consistently significantly enriched relative to mainland and non-bird islands, a relationship expected due to the progressive volatilization of ^14^N rich ammonia from decomposing guano deposits. Our findings demonstrate that seabird-mediated marine nutrient deposits provide the source for solubilized nitrogen on desert islands, which stimulate terrestrial plant production in the cardón cactus beyond that seen in either mainland ecosystems or non-seabird islands.

## Introduction

The land-sea interface is distinguished by near constant movement of materials between terrestrial and marine biomes. Allochthonous subsidies, originating in one location and transported to a disparate environment, often take the form of marine nutrients. These marine derived subsidies, when delivered to coastal regions or islands, can provide nutrient inputs that impact the functioning of terrestrial ecosystems^1–3^. The iconic example of the decaying bodies of spawning salmon enhancing soil profiles and terrestrial plant biodiversity illustrates the importance of these multi-step trophic land-sea linkages^4–6^.

Globally, coastal areas represent only 8% of total land area but provide 20% of oceanic production^7^. Seabirds are present in a wide range of high productivity marine systems around the world and are essential to nutrient cycling between land-sea systems. They facilitate the exchange of nitrogen and other chemical subsidies between the two biomes^8–12^ and alter physical and soil conditions directly affecting plant richness, production, and distribution^1,4,13–15^. Seabird guano, which is deposited on land in coastal regions or island systems, as well as seabird bodies themselves (dead adults, chicks, and eggs) are prime examples of land-sea connectivity.

In the San Juan archipelago, seabird-derived guano in intertidal regions appeared to increase the abundance of certain algal species while simultaneously decreasing intertidal plant biodiversity^16^. Seabirds have been shown to increase native tree abundance and soil nitrogen where they roost on tropical islands^17^. In coastal areas where native plants create poor bird habitat, nutrient cycling is limited due to decreased seabird guano deposits^18^. In Antarctica, penguin rookeries classically exhibited significant nitrogen deposition to the island ecosystem^8,19,20^. Similarly, in the Gulf of California, previous work has shown areas of seabird influence possessed increased nitrogen content and unusually elevated nitrogen stable isotope ratios that are characteristic of guano deposition and diagenesis^12,21^.

The arid islands in the Gulf of California, Mexico are one of the best manifestations of the effects of marine nutrient cycling to terrestrial systems. Bird islands—typically small (< 3 km^2^), predator free, with low topography, that occur in high productivity waters—are utilized for habitat by a large quantity of seabirds. All islands and the adjacent Baja California peninsula and Sonoran mainland also host the widespread Sonoran Desert columnar cactus *Pachycereus pringlei* (S. Watson) Britton & Rose (*cardón* or *sahueso*, Cactaceae). This paper examines the role of seabird influence on desert islands by looking at the path of nitrogen to the cardón cactus across the entire Gulf of California region.

Due to high perimeter-to-area ratios, small islands are disproportionally influenced by the surrounding marine environment^22^. Guano excreta on bird islands is a significant component of both island appearance and island function, with bright white guano crusted over rock surfaces once as thick as 10 cm in some places^23^. A variety of bird species are known to frequent bird islands; in the Gulf of California, species with the highest abundances include Heermann’s Gulls, Elegant Terns, Blue-footed Boobies, and Western Gulls^24^.

**Figure 1:**
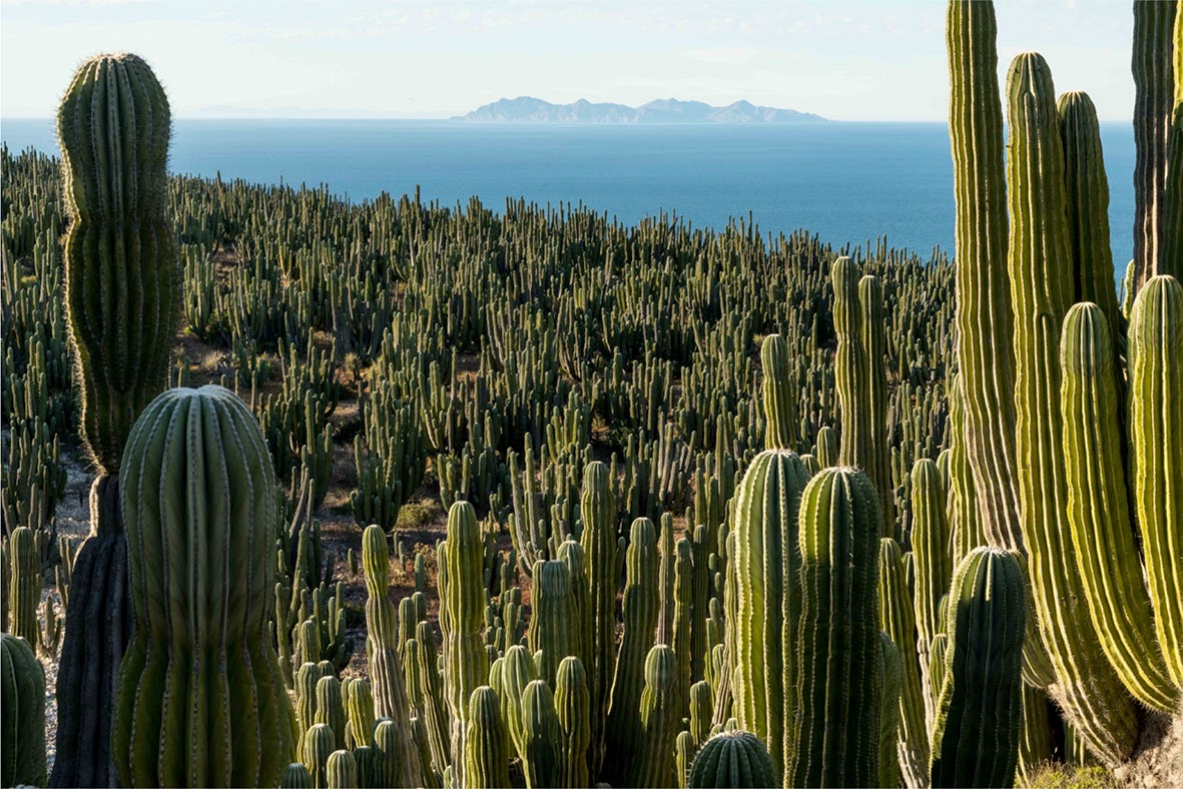
View from summit of Isla San Pedro Mártir, showing the cardón cactus forest. © John Sherman.

These bird islands have also been shown to have significantly reduced plant diversity, likely due to the higher nitrogen and phosphorus nutrient concentrations that act as a filter that exclude the presence of many plant species^25^, which has also been seen in other bird island systems^26^. However, cacti are seen to occur in both higher diversity and abundance on bird islands in the Gulf of California^27^. Indeed, several islands are distinguished by dense forests of cardón, which cover the island and contribute a large amount of plant biomass in such an arid setting^27^ (Fig. 1). What then is the role of the marine nutrients on the structure of these island communities?

Guano contains nitrogen in the form of NH_4_^+^, NO_3_^-^, and as a component of uric acid (C_5_H_4_N_4_O_3_). Each of these undergoes chemical alteration at different rates of decomposition^19^. Previous research has demonstrated that the nitrogen isotope composition of bulk guano-enriched soils is significantly elevated due to the volatilization of ammonia, leaving the remaining material with more positive δ^15^N values^8^. The breakdown of uric acid releases large amounts of ammonia, with an associated large isotopic fractionation between ammonia and the remaining nitrogen salts. This leads to the loss of a large amount of nitrogen in a form that favors ^14^N, and leaves the soil zone enriched in ^15^N. This process follows a Rayleigh fractionation relationship leading to unusually positive δ^15^N values in the soil^28^. Guano-enriched soils have much higher nitrogen isotope ratios than non-guano-enriched areas^13^ and create spatial variation in the chemical soil composition of island systems^11^.

In years of higher precipitation such as El Niño years in Southwest North America or when hurricanes reach this region, there is an elevated presence of decomposed guano due to the mobilization of nutrients from the increased moisture. During these pulse years, the soil exhibited increased nitrogen levels with increased plant productivity^13^. It is suggested that guano deposition in terrestrial ecosystems could increase soil nitrogen content by up to 100 times its original amount^9^. Plants growing in areas with higher guano concentrations took up nitrogen sourced in guano and as a result had significantly elevated δ^15^N values^2,11,12,29^. In natural soils and plants, ones not affected by artificial fertilizers, nitrogen isotope ratios that are higher than + 25‰ (ATM) are rare unless they are from systems that have significant guano input^28,30^. These elevated δ^15^N values therefore become a tracer for the impact of seabird guano in the plant community.

We used nitrogen stable isotope ratios to (A) see if there is a trophic linkage that originates in the ocean, is linked to land by seabirds and their guano, deposited into the soil, and then utilized by the tissue of the cardón on the representative island of San Pedro Mártir, and (B) assess if this nutrient transfer is a manifestation of sea-to-land connections on seabird islands that is not found in mainland habitats or islands without seabird colonies by looking at the whole of the Gulf of California region and its islands.

## Results

**Figure 2:**
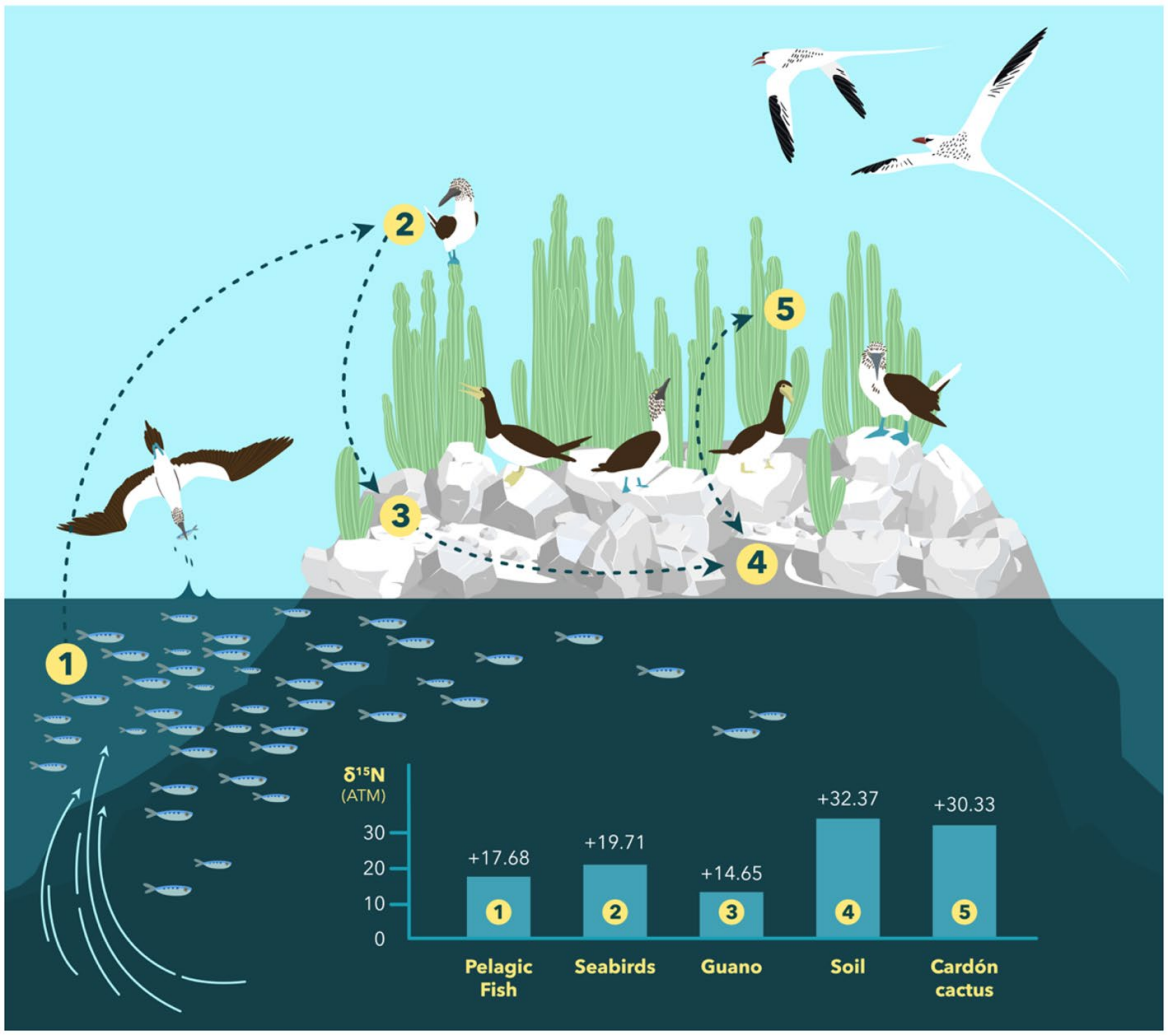
Trophic nutrient transfer across the land-sea interface of Isla San Pedro Mártir. Diagram shows the flow of nitrogen sourced in upwelling in the Gulf of California as traced by stable nitrogen isotope values. (1) Pelagic fish that are eaten by (2) seabirds that (3) deposit guano on bird islands, which (4) enter the island soil that is then (5) utilized by the cardón cactus, which returns to the soil upon the death of the cactus. Figure by Paola Ramirez.

Nitrogen stable isotope values showed a trophic linkage from the sea onto the land on Isla San Pedro Mártir and uniquely elevated soil and cactus δ^15^N values on bird islands relative to all other regions where the cardón cactus occurs (Fig. 2; Table 1). On Isla San Pedro Mártir, fish samples generally had a mean nitrogen stable isotope value of + 17.7 ± 0.88, and seabird feathers displayed δ^15^N values with a mean of + 19.7 ± 0.67 (all nitrogen isotope values are reported in ‰ units relative to the ^15^N/^14^N ratio of atmospheric nitrogen, ATM). Fresh guano deposited by seabirds on the island had a mean δ^15^N value of approximately + 14.8 ± 2.12. We presume that this guano, as it ages, follows the commonly observed diagenetic pathway of microbial degradation into ammonia and solid ammonium salts and nitrates^28^. The volatilization of ammonia gas disproportionally removes ^14^N from the guano, leaving the remaining guano in the soil enriched in ^15^N, with δ^15^N values averaging + 32.4 ± 3.55 on the island. Mártir cardón specimens had an average δ^15^N value of + 30.3 ± 3.86. Other seabird islands in the Midriff region (Islas Alcatraz, Las Ánimas, Cardonosa, Cholludo, Partida, Rasa, and Salsipuedes) showed consistently high soil and cardón stable nitrogen isotope values, with an average of + 34.3 for soil and + 30.3 for cacti tissue (Table 2).

**Table 1:** Results of Tukey HSD multiple comparisons of means test between the four regions (bird island, non-bird island, Baja California, and Sonora) for δ^15^N values for soil and cardón.

Regions compared	diff	lower CI	upper CI	p_adj_
**Soil**				
Non-bird island—Bird island	− 17.42	− 23.75	11.09	< 0.0001
Bird island—Baja California	22.98	16.93	29.03	< 0.0001
Sonora—Bird island	− 20.56	− 29.38	− 11.75	< 0.0001
Non-bird island—Baja California	5.56	− 0.29	11.41	0.07
Sonora—Non-bird island	− 3.14	− 11.82	5.54	0.76
Sonora—Baja California	2.42	− 6.07	10.90	0.86
**Cardón**				
Non-bird island—Bird island	− 16.58	− 23.07	− 10.09	< 0.0001
Bird island—Baja California	22.12	15.91	28.32	< 0.0001
Sonora—Bird island	− 19.05	− 28.10	− 10.10	< 0.0001
Non-bird island—Baja California	5.54	− 0.47	11.54	0.08
Sonora—Non-bird island	− 2.48	− 11.38	6.43	0.87
Sonora—Baja California	3.06	− 5.64	11.76	0.77

diff is the difference between groups and the lower and upper CI reflects the 95% family-wise confidence levels.

**Table 2:** Average δ^15^N values for Isla San Pedro Mártir and the four sample regions.

Sample	N	δ^15^N	S.D	Range
**Isla San Pedro Mártir**				
Fish	11	+ 17.7	0.88	+ 16.6–19.3
Seabird feather	16	+ 19.7	0.67	+ 18.2–20.5
Guano	13	+ 14.7	2.26	+ 11.4–20.3
Soil	20	+ 32.4	3.55	+ 25.9–37.9
Cardón tissue	20	+ 30.3	3.86	+ 25–37.6
**Bird islands **^**1**^				
Soil	85	+ 34.3	3.84	+ 24.3–48.0
Cardón tissue	85	+ 30.3	3.44	+ 21.1–38.7
**Non-bird islands **^**2**^				
Soil	74	+ 15.0	3.40	+ 9.0–25.1
Cardón tissue	74	+ 11.7	2.26	+ 4.3–20.8
**Mainland Sonora**				
Soil	30	+ 13.8	2.01	+ 10.3–21.1
Cardón tissue	30	+ 11.2	1.88	+ 7.8–14.9
**Baja California peninsula**				
Soil	109	+ 11.4	2.92	+ 5.5–18.9
Cardón tissue	110	+ 8.1	3.12	+ 2.5–17.3

^1^Bird island values include Isla San Pedro Mártir.

^2^Non-bird island values exclude Isla San Diego.

**Figure 3:**
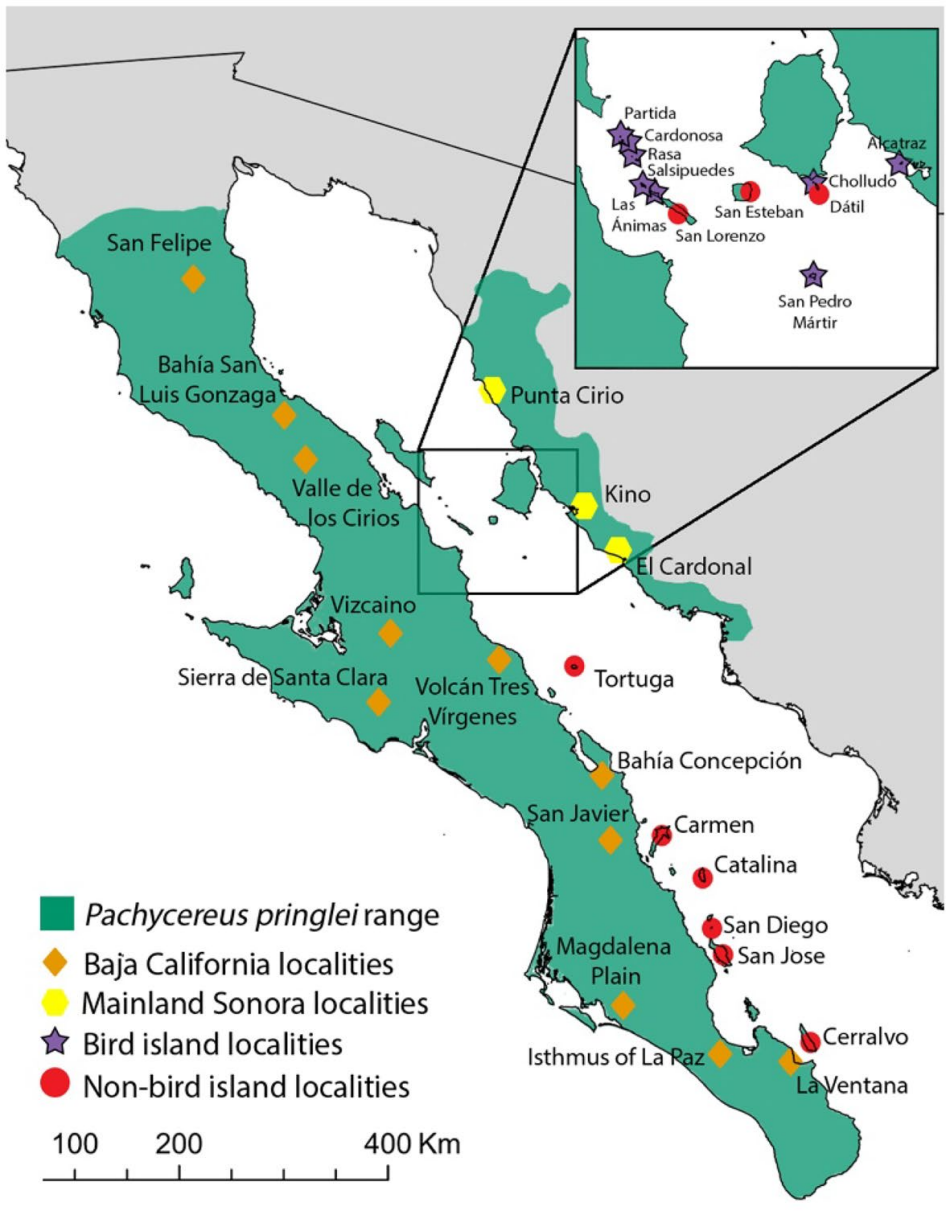
Sampling localities across the range of cardón. Figure by Wilder in Photoshop.

**Figure 4:**
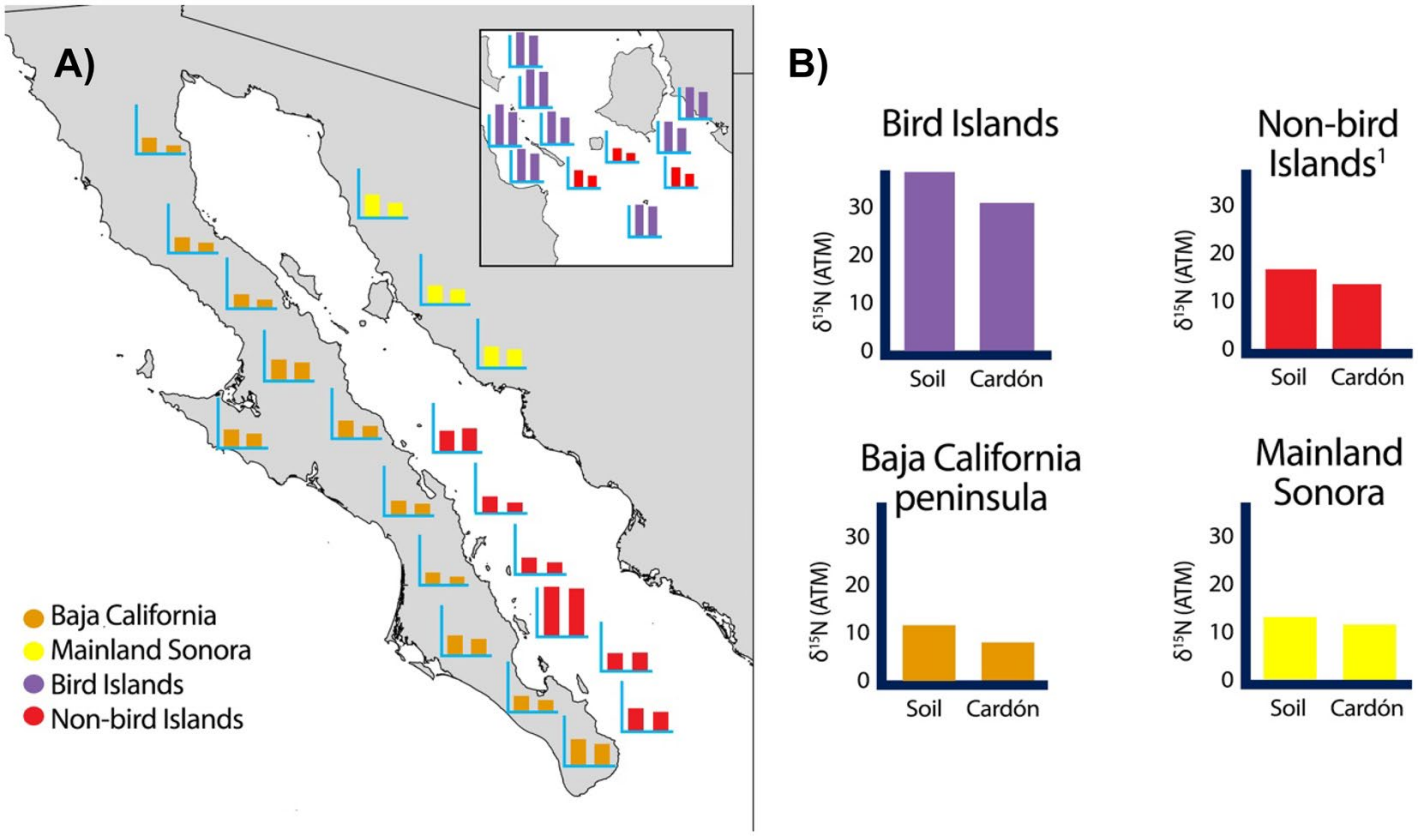
Average δ^15^N values for soil and cardón across sampling localities. (**A**) Geographic distribution of soil and cardón δ^15^N values at each sampling site. (**B**) Comparison of soil and cardón δ^15^N values for bird islands, non-bird islands (^1^excluding Isla San Diego), Baja California, and Sonora. Figure by Wilder in Photoshop.

Soil and cactus δ^15^N values differed among regions (one-way ANOVA, soil: F(3, 27) = 38.92, p < 0.0001; cardón: F(3, 27) = 33.86, p < 0.0001). Further, soil and cardón stable nitrogen isotope values were consistently higher and more stable on bird islands compared to other sites (Figs. 3, 4, and Table 1). Average nitrogen isotope values for peninsular populations of soil were + 11.4 and + 8.13 for cacti tissue. Likewise, the non-bird Gulf islands average values per population for soils was + 15.0 and + 11.7 for cardón cacti. One non-bird island, Isla San Diego, had significantly higher δ^15^N values of + 34.0 ± 1.89 and + 32.8 ± 2.67 for soil and cardón respectively and is reported separately from the other non-bird islands. Mainland Sonora populations had average values of + 11.4 for soil, and + 11.2 for cacti (Table 2, Supplemental Table S1).

As expected, δ^15^N values for soil and cardón were elevated on seabird islands relative to other sites, but values did not differ with distance from the mainland or island type (oceanic or land bridge) (linear mixed models, soil: beta = 9.54, t = 9.76, p < 0.0001; cardón: beta = 9.34, t = 2.78, p = 0.0146). Elevated δ^15^N values disproportionately occurred on small islands relative to larger ones, indicating an effect of area (linear mixed models, soil: beta = − 2.45, t = − 3.73, p = 0.0025; cardón: beta = − 2.25, t = − 3.06, p = 0.0086; Supplemental Fig. S1; Supplemental Tables S2 and S3).

Soil nitrogen content differed across sampling regions (one-way ANOVA, F(3, 27) = 10.1, p < 0.0001) and was higher on bird islands (average = 0.55 wt%) than soil located on non-bird islands or non-bird mainland locations (average = 0.11 wt%, Supplemental Fig. S2). Other regional differences were not significant. Cardón samples from bird islands were not statistically different from non-bird populations in organic nitrogen content (Supplemental Fig. S3).

## Discussion

Nutrient subsidies are known to supply otherwise unavailable resources that create unique growing conditions in diverse habitats around the world. Seabirds are an important vector, which have large population densities at oceanic sites of high primary productivity, often associated with upwelling. Zones of upwelling often correspond with arid or mediterranean-type climates on the western side of continents^31^. Accordingly, the effect of seabird-transmitted marine nutrients in shaping vegetation patterns can be overlooked due to relatively modest and arid-adapted floras. Yet, columnar cactus diversity is high in the Sonoran Desert of the Gulf of California^32^ with one such species the cardón, *Pachycereus pringlei*, ubiquitous on the Gulf islands and the adjacent mainland and Baja California peninsula. Within this context, several islands in the Gulf of California stand apart, covered in a green forest of columnar cacti, unrivaled in density (Fig. 1). What creates these cactus forests? This study helps elucidate the role of seabirds in bridging the land-sea boundary, with elevated δ^15^N values on seabird islands indicating the importance of marine nutrients shaping terrestrial ecosystems.

### Land-sea interface in the Gulf of California

Strong winter and spring winds from the northwest, year-round tidal fluctuations, and constriction points between the Midriff Islands cause coastal-trapped waves that lead to mixing and upwelling that stimulate globally high values of primary productivity in the Gulf of California (rates can exceed 4 g C/m^2^/day^33,34^). The persistent cold-water upwelling in combination with the arid climate of the Sonoran Desert leads to a marked aridity on the islands. Upwelling of cold water with nitrogen-rich nutrients provides food for coastal fish species, who are then consumed by resident and semi-resident island bird populations, as well as numerous marine mammal and fish species^35,36^. These birds deposit guano onto island habitats, and as nutrients from guano leach into the soil, they are then able to be utilized by cacti and annual plant species on the island (Fig. 2).

The flow of nutrients from marine to terrestrial environments has been well documented on small islands in the Gulf of California, both in the form of oscillating land-driven (wet, El Niño years) or sea-based (dry, La Niña and most years) trophic linkages^37^, and by nesting seabirds^10,11^. We can now extend this chain of interactions to the region's anomalously dense cactus forests.

As shown in previous island biogeographic analyses for these islands^25^, isolation did not control plant species diversity, and the degree of isolation did not correlate with our cardón δ^15^N results. The presence of the Mexican mainland and the Baja California peninsula along the length of the Gulf maintains relative close proximity between these two land masses, the distance between which plant dispersal syndromes can usually overcome. Likewise, seabird islands are found on both oceanic and land bridge islands (islands previously connected to the mainland). Importantly however, seabird islands are small islands with high perimeter to area ratios that allow the influx of marine resources and good nesting habitat.

The cactus forest on Isla San Pedro Mártir has a density of ca. 2700 plants/ha^38^ and a neighboring bird island, Isla Cholludo, has an order of magnitude more cardón cacti with ca. 23,500 plants/ha (Wilder unpublished data). These densities are far greater than the rest of the species' range (ca. 150 plants/ha in Baja California, ca. 60 plants/ha in Sonora;^39^) and are likely some of the densest, if not the densest, columnar cacti populations in the world^32^. In addition to the role of nitrogen subsidies from guano, the increased abundance of cardón cacti on the islands is thought to be due to the absence of native rodents and other terrestrial herbivores that are known to prey on seedlings and young columnar cacti, as well as a favorable maritime climate^38,40^. The large juicy fruit of this columnar cactus, seasonally abundant on the island in the spring, is an important resource for the island's terrestrial biota. As these cacti mature on the island, they tend to topple, potentially because of strong ocean winds and occasional tropical storms and their position in a loose, rocky substrate^38^. Their decomposition propagates the nutrient cycle.

Nitrogen stable isotope ratios clearly set the bird islands apart from other locations around the Gulf of California due to the unusual isotopic signature of decaying seabird guano. Because the elevated δ^15^N values are the result of breakdown of seabird guano, the nitrogen isotope ratios are evidence of the transfer of large amounts of nitrogen from the marine to island setting. Our data revealed a large difference in δ^15^N values between bird islands, compared to non-bird island, mainland, and peninsular populations, with higher nitrogen isotope ratios present in bird islands compared to the other localities (Fig. 4; Table 1; Supplemental Figs. S2 and S3).

One island sampled, San Diego, also exhibited higher δ^15^N values (+ 34.0 for soil and + 32.8 for cardón) on par with those of bird islands. San Diego does not demonstrate direct bird guano influence as Mártir does, but observations at the study site did find seabird bone remains at the base of several cardón cacti sampled. It is possible that seabirds maintain an influence on this island via nesting, death, and decomposition at the base of the cacti that permits elevated δ^15^N values, uncharacteristic of non-bird guano island systems. Nutrient cycling on San Diego island, as well as an elevated floristic diversity relative to the island's size (E. Ezcurra personal communication), indicate that this island deserves further study.

### Lessons from nitrogen

The patterns present on seabird islands in the Gulf of California are consistent with previous research; systems subsidized with external nitrogen deposits tend to include fewer species^11,16,26,41^. Species richness and type can be impacted by spatial subsidies^42^. Plants that have high rates of resource acquisition over a short period of time have been shown to be associated with seabirds on islands^11,43^. It has also been shown that plants on subsidized island systems possess different physiological mechanisms, including conservative water use strategies instead of higher photosynthetic rates in annual species to manage increased resources and growth during inter-pulse periods^13^. It is possible that cacti and other organisms present on seabird islands with high, “toxic” levels of guano necessitate the development of physiological tolerances that allow rapid growth and water conservation in conditions of limited water and unlimited nutrients. Increased guano deposition on the seabird islands of the Midriff region delivers nutrients that may be used uniquely by cardón cacti (and cholla cacti on some islands, e.g., Rasa) and seasonal annuals^13^ to increase their population density in a way that is not seen in other island plant species.

Increased nitrogen deposits have shown both beneficial and limiting impacts on plant species^27,44^. Cacti utilize nitrogen in a variety of ways, such as components of chlorophyll and nucleic acids, and as a factor in protein synthesis. When there is a greater store of available nitrogen during development, cacti exhibit an increase in soil root growth and shoot growth^44^. Plants with low relative growth rates are known to have a high capacity to store sporadically available nutrients^21^.

The nitrogen isotope values obtained on Midriff seabird islands are some of the highest reported for any region globally and are consistent with previous research in this region. Previously, the highest δ^15^N value (that we have located) for a plant in a natural environment outside the seabird islands of the Gulf of California was + 21.4 from a prairie wildflower (*Callirhoe involucrata*, Malvaceae) in a tallgrass prairie^45,46^. Schoeninger & DeNiro^47^ likewise did not find δ^15^N values higher than + 20.0 for plants, although they report δ^15^N values for marine fish (+ 11.1 to  + 16.0) and seabirds (+ 9.4 to + 17.9), the higher values of which compare well with our results. Studies in the Gulf of California islands in the vicinity of Bahía de los Angeles by Stapp et al.^12^ similarly compare well to the δ^15^N values we obtained for guano from Isla San Pedro Mártir of + 14.77 ± 2.26 (their values, + 14.2 ± 2.34) as well as our average bird island δ^15^N soil values + 34.3 ± 3.84 (their values, + 28.3 ± 5.44).

Because plants take on the nitrogen isotope ratio of the available nitrogen in the soil with only minor modification of the δ^15^N values, we expect that any plants growing on guano fertilized soils will take on the highly elevated δ^15^N values characteristic of these environments^30,48,49^. Enriched δ^15^N values in plants can correspond with high productivity or fertilized habitats (agriculturally and ecologically), largely due to ammonia volatilization^48^. These results further confirm that guano serves as excellent fertilizer, which was harvested from many of the bird islands of the Gulf of California in the late 1800s^50^.

In all bird islands, a significant increase in the mean δ^15^N values occured between seabird guano and soil. Other studies have also observed this increase in δ^15^N soil values relative to the source guano, arguing that the volatilization of ammonia during diagenesis of these nitrogen-rich deposits^8,29^ leads to the high δ^15^N soil values. Our data demonstrated the trophic level increases in marine-sourced nitrogen isotope values as well as the notable diagenetic increase from guano to soil and cardón samples (Table 1). Our data also recorded a consistent decrease in δ^15^N values from soil to cactus tissue of about 2.6–4.0 ‰. Although the causes of this decrease in δ^15^N values are not well understood, this is in agreement with most plant tissues, which have slightly lower δ^15^N values than the soil on which they grew^46,49^. Nitrogen isotope ratios can show high variance in systems where isotopic fractionation involves significant losses of volatile nitrogen as ammonia^6,12,29,51^.

Seabird island soils were enriched in nitrogen content compared to non-bird islands, most likely due to the guano subsidy (Fig. S2). This observation is consistent with prior data from the region^11^. However, we did not find evidence for a significantly higher nitrogen content in the bird island cardón cactus tissue (Fig. S3). The cardón tissue measured across all sites is the green photosynthesizing cortex immediately beneath the epidermis of the cacti. Perhaps the nitrogen content of these tissues is controlled more by the needs of photosynthesis rather than nitrogen availability.

Other terrestrial systems at the land-sea interface, such as penguin rookeries, display a similar mechanism of marine to terrestrial nutrient transfer where a large isotopic fractionation in nitrogen isotope ratios in decaying guano is evident^8,19^. Guano deposits onto soil substrate allow uric acid to leech into soil and decompose. A study by Mizutani et al.^20^ incubated rookery soil with added uric acid. In 10 days, the decay of the uric acid and volatilization and loss of ammonia led to an 8.4 ‰ increase in δ^15^N values of the remaining soil nitrogen. A similar process is likely present on the bird islands of this study.

## Conclusion

The enriched δ^15^N values uniquely exhibited in soil and cardón cacti on bird islands in the Gulf of California are established by marine nitrogen subsidies deposited via seabird guano and its subsequent decay. The ammonia-rich guano accumulated on these desert islands, once the focus of extensive commercial harvesting, is a byproduct of regional marine productivity. Nitrogen cycling mediated by seabirds moves nutrients from marine to terrestrial biomes, significantly affecting terrestrial plant production—in this case providing resources for some of the densest columnar cactus populations in the world, a nutrient relationship that crosses the land-sea boundary.

## Methods

### Study system

The Gulf of California, also known as the Sea of Cortés, is an ecologically and geologically diverse region^52^. The Gulf has separated the Baja California peninsula and mainland Mexico for about six million years^52^, housing numerous islands and an array of both endemic and migratory species of marine organisms, mammals, plants, insects, and birds^53^. Gulf of California islands have diverse geological histories and run the spectrum of island biogeographic variables in scale, isolation, and age^25,53^. The 17 islands sampled in this study, including the eight bird islands, encompass land bridge and oceanic islands and islands close to and far from shore.

Seabirds thrive in the Gulf, where they have access to nutrient-rich fishes, products of high productivity upwelling in the waters around islands^10^. This high productivity is especially concentrated in the Midriff region of the Gulf of California, a desert archipelago that stretches west to east at ca. 29° latitude. While present in other regions of the Gulf, the Midriff region contains the greatest number of seabird islands and nearly all seabird islands with cardón cacti and any floristic diversity. This study focused in part on Isla San Pedro Mártir, which is one of the most important seabird rookeries in Mexico, as a model system for the seabird islands and the trophic linkages described. Eighty-five species of birds have been recorded on this island, including eight breeding seabirds^54^. The colonies of the Brown Booby (*Sula leucogaster*) and Blue-footed Booby (*S. nebouxii*) are among the world’s largest and are seasonally the most abundant birds on the island, and the colonies of the Brown Pelican (*Pelecanus occidentalis*) and Red-billed Tropicbird (*Phaethon aethereus*) are among the largest in Mexico^55^. Isla San Pedro Mártir’s substrate is coated with guano and contains a flora of 28 species^38,56^ with plant biomass dominated by the cardón cactus (Fig. 1) and seasonally abundant ephemerals post-El Niño or hurricane-derived storms. Designation of Gulf of California islands as seabird or not follows Velarde et al.^24^ and Wilder et al.^25^.

### Sample collection

Sample collection took place at two scales. Five steps of the land-sea trophic chain were sampled on Isla San Pedro Mártir in November 2017: fish, seabirds, seabird guano, soil, and cardón cactus tissue. The samples from this island are representative of fish, seabird, and guano found on other seabird islands in the Midriff region based on the proximity to and the consistency of marine conditions and land-sea dynamics in this area (e.g., Refs.^12,25^). Fish were sampled by collecting freshly regurgitated pelagic fish (sardines) from the blue-footed booby. Regurgitation is a common feeding behavior of this species. Seabirds were sampled through collection of recently shed feathers from the blue-footed booby, which nest adjacent to the cardón cacti and are a dominant source of the guano on the island (N = 16). Guano, derived from either the blue-footed or brown booby (N = 13), was collected by scraping recent excrement off rocks and camping material. The study passively collected excreted guano, dropped feathers from the birds, and excreted fish thrown up by the birds as part of their normal behavior. No experiments were conducted with live vertebrates that needed approval by a named institutional and/or licensing committee. Soil samples were gathered adjacent to the root and at the base of individual cardón cacti sampled for δ^15^N analysis by scraping off the top couple centimeters of soil and excavating to ca. 15 cm belowground (N = 20). Cardón tissue was collected from a small section of a single rib per individual on a stem with recent growth (N = 20). Soil, guano, and feather samples were stored dry at ambient temperature. Fish samples were stored on ice until they could be frozen (after approximately 24 h) for transport to the lab. Cardón tissues were dried with absorbent paper in a plant press to remove tissue moisture and reduce bacterial degradation.

For comparison, soil and cardón tissue were sampled throughout the range of the species, including other islands of the Gulf of California, both seabird (8, including Isla San Pedro Mártir) and non-bird islands (9), along the Baja California peninsula (11) and in Sonora, MX (3) in 2018 and 2021. In total, 10 soil and 10 cardón collections were made from each of 31 locations with 298 soil samples and 299 cactus samples in total (Fig. 3; Supplemental Table S4).

### Lab analysis

Cardón tissue for isotope measurement was limited to the chlorenchyma tissue immediately below the epidermis. Soil samples were prepared by measuring the organic matter contained in the very fine particles in the soil sample containers, which presumably allowed us to bias the sample toward an organic matter contributed by a variety of organisms in the landscape and soil zone. We wanted to avoid a sample dominated by only a few large organic particles, perhaps from a single plant. Fish samples were collected from muscle tissue and dried overnight in a 50 °C oven. Dirt was removed from the bird feathers with soap and water, and feathers were dried. Both fish samples and bird feathers were de-oiled using a 2:1 mixture of chloroform:methanol and samples were again dried overnight. De-oiling is a standard practice in the lab, and apparently was not needed when working with the nitrogen isotope ratio measurement of these animal tissues. Note that it does not affect the δ^15^N results—see the discussion of lipid removal, δ^13^C, and δ^15^N values in Post et al.^57^ and Kiljunen et al.^58^.

Samples were analyzed in the Environmental Isotope Laboratory in the Geosciences Department of the University of Arizona. The stable nitrogen isotope ratio (δ^15^N) and nitrogen content were measured on a continuous-flow gas-ratio mass spectrometer (Finnigan Delta PlusXL). Samples were combusted using an elemental analyzer (Costech) coupled to the mass spectrometer. Standardization is based on acetanilide for elemental concentration, IAEA-N-1 and IAEA-N-2 for δ^15^N. Precision is better than ± 0.15 for δ^15^N (1σ), based on repeated internal standards.

### Statistical analysis

We tested for among-site differences in soil and cactus δ^15^N values using one-way ANOVAs. We then tested for pairwise differences amongst all combinations of regions (bird island, non-bird island, Baja California, Sonora) in soil and cactus δ^15^N values using Tukey's HSD.

Similarly, we used one-way ANOVAs to look for among-site differences in soil and cactus nitrogen content. We then tested soil nitrogen content (cactus was non-significant) for pairwise difference amongst all combinations of regions (bird island, non-bird island, Baja California, Sonora) using Tukey's HSD.

To see if the island biogeographic variables of area, seabird or non-seabird islands, distance from mainland, and type of island (oceanic or land bridge) accounted for patterns in the δ^15^N values on the Gulf of California islands we used linear modeling. We ran a linear mixed-effects model^59^ with site as a random effect, and bird island or non-bird island, area, and island type as fixed effects for both soil and cardón cactus in R^60^. This model had the form:

$${\text{N}}\sim {\text{B}}_{0} + {\text{B}}_{{1}} \times {\text{BirdIsland }} + {\text{ B}}_{{2}} \times {\text{Area }} + {\text{ B}}_{{3}} \times {\text{Islandtype}} + \gamma + \varepsilon$$

where N is the δ^15^N value, BirdIsland is whether the island is a seabird island or a non-bird island, area is the size of the island, Islandtype is if the island is of oceanic or land bridge origin, γ is the random intercept effect of site, and ε is the residual error.

## Supplementary Information

Supplementary Information 1.

Supplementary Information 2.

## Data Availability

All data generated or analyzed during this study are included in supplementary information files of this published article.
