# Supplementary material for: Marine subsidies produce cactus forests on desert islands
Source: Sci Rep. 2022 Oct 12;12:17110. doi: 10.1038/s41598-022-21133-3 (PMC9556768; doi:10.1038/s41598-022-21133-3)
Supplement: Supplementary file 2 — Supplementary Information 2. [file 41598_2022_21133_MOESM2_ESM.docx]

**Marine Subsidies Produce Cactus Forests on Desert Islands**

**Supplementary Material**

**Figure S1.** Relationship of log area of the Gulf of California islands and δ^15^N values for soil and cardón samples. Seabird islands (light blue) and non-bird islands (red). Note, the high δ^15^N non-bird island value is Isla San Diego.

**Figure S2.** Soil nitrogen content relative to soil δ15N across regions.

**Figure S3.** Cardón N content relative to cardón δ15N across regions.

**Table S1.** Average δ^15^N values for soil and cardón samples from all sites.

| **Name** | **Location** | **Bird island?** | **Soil or cardón** | **N** | **Average d15N**  **(‰ ATM)** | **Standard deviation** |
| --- | --- | --- | --- | --- | --- | --- |
| Bahía Concepción | Baja California peninsula | no | cardón | 10 | 7.2 | 1.29 |
| Bahía San Luis Gonzaga | Baja California peninsula | no | cardón | 10 | 6.3 | 1.41 |
| Isthmus of La Paz | Baja California peninsula | no | cardón | 10 | 7.5 | 0.81 |
| La Ventana | Baja California peninsula | no | cardón | 10 | 14.2 | 1.96 |
| Magdalena Plain | Baja California peninsula | no | cardón | 10 | 10.3 | 2.30 |
| San Felipe | Baja California peninsula | no | cardón | 10 | 5.2 | 1.71 |
| San Javier | Baja California peninsula | no | cardón | 10 | 5.0 | 1.38 |
| Sierra de Santa Clara | Baja California peninsula | no | cardón | 10 | 8.8 | 1.44 |
| Valle do los Cirios | Baja California peninsula | no | cardón | 10 | 5.7 | 1.74 |
| Vizcaino | Baja California peninsula | no | cardón | 10 | 11.4 | 1.03 |
| Volcán Tres Virgenes | Baja California peninsula | no | cardón | 10 | 7.9 | 1.71 |
| Isla Carmen | Gulf of California | no | cardón | 5 | 11.0 | 0.77 |
| Isla Catalina | Gulf of California | no | cardón | 10 | 7.7 | 2.16 |
| Isla Cerralvo | Gulf of California | no | cardón | 10 | 12.5 | 3.29 |
| Isla Dátil | Gulf of California | no | cardón | 10 | 14.5 | 2.74 |
| Isla San Diego | Gulf of California | no | cardón | 10 | 32.8 | 2.67 |
| Isla San Esteban | Gulf of California | no | cardón | 10 | 9.1 | 1.41 |
| Isla San Jose | Gulf of California | no | cardón | 10 | 12.1 | 0.98 |
| Isla San Lorenzo | Gulf of California | no | cardón | 10 | 13.6 | 2.55 |
| Isla Tortuga | Gulf of California | no | cardón | 9 | 12.6 | 2.43 |
| Isla Alcatraz | Gulf of California | yes | cardón | 10 | 27.9 | 1.74 |
| Isla Cardonosa | Gulf of California | yes | cardón | 10 | 34.9 | 1.63 |
| Isla Cholludo | Gulf of California | yes | cardón | 10 | 26.1 | 2.36 |
| Isla Las Ánimas | Gulf of California | yes | cardón | 10 | 28.8 | 2.14 |
| Isla Partida | Gulf of California | yes | cardón | 10 | 32.2 | 0.71 |
| Isla Rasa | Gulf of California | yes | cardón | 5 | 26.9 | 1.88 |
| Isla Salsipuedes | Gulf of California | yes | cardón | 10 | 34.8 | 2.72 |
| Isla San Pedro Mártir | Gulf of California | yes | cardón | 20 | 30.3 | 3.85 |
| El Cardonal | Sonora | no | cardón | 10 | 11.9 | 1.31 |
| Kino | Sonora | no | cardón | 10 | 9.4 | 1.03 |
| Punta Cirio | Sonora | no | cardón | 10 | 12.3 | 1.81 |
| Bahía Concepción | Baja California peninsula | no | soil | 10 | 9.4 | 1.33 |
| Bahía San Luis Gonzaga | Baja California peninsula | no | soil | 9 | 9.8 | 1.16 |
| Isthmus of La Paz | Baja California peninsula | no | soil | 10 | 10.4 | 0.76 |
| La Ventana | Baja California peninsula | no | soil | 10 | 17.3 | 1.10 |
| Magdalena Plain | Baja California peninsula | no | soil | 10 | 13.7 | 1.61 |
| San Felipe | Baja California peninsula | no | soil | 10 | 10.2 | 1.94 |
| San Javier | Baja California peninsula | no | soil | 10 | 7.7 | 1.22 |
| Sierra de Santa Clara | Baja California peninsula | no | soil | 10 | 11.5 | 0.94 |
| Valle de los Cirios | Baja California peninsula | no | soil | 10 | 8.9 | 1.18 |
| Vizcaino | Baja California peninsula | no | soil | 10 | 13.9 | 1.05 |
| Volcán Tres Virgenes | Baja California peninsula | no | soil | 10 | 12.2 | 1.44 |
| Isla Carmen | Gulf of California | no | soil | 5 | 14.1 | 1.13 |
| Isla Catalina | Gulf of California | no | soil | 10 | 11.0 | 1.35 |
| Isla Cerralvo | Gulf of California | no | soil | 10 | 14.9 | 3.70 |
| Isla Dátil | Gulf of California | no | soil | 10 | 20.3 | 1.95 |
| Isla San Diego | Gulf of California | no | soil | 10 | 34.0 | 1.89 |
| Isla San Esteban | Gulf of California | no | soil | 10 | 13.5 | 1.50 |
| Isla San Jose | Gulf of California | no | soil | 10 | 11.9 | 1.41 |
| Isla San Lorenzo | Gulf of California | no | soil | 10 | 19.9 | 3.03 |
| Isla Tortuga | Gulf of California | no | soil | 9 | 14.6 | 3.08 |
| Isla Alcatraz | Gulf of California | yes | soil | 10 | 31.5 | 3.51 |
| Isla Cardonosa | Gulf of California | yes | soil | 10 | 38.6 | 1.85 |
| Isla Cholludo | Gulf of California | yes | soil | 10 | 30.8 | 2.96 |
| Isla Las Ánimas | Gulf of California | yes | soil | 10 | 32.0 | 3.63 |
| Isla Partida | Gulf of California | yes | soil | 10 | 36.4 | 1.54 |
| Isla Rasa | Gulf of California | yes | soil | 5 | 32.1 | 1.24 |
| Isla Salsipuedes | Gulf of California | yes | soil | 10 | 41.1 | 3.37 |
| Isla San Pedro Mártir | Gulf of California | yes | soil | 20 | 32.3 | 3.55 |
| El Cardonal | Sonora | no | soil | 10 | 13.6 | 0.89 |
| Kino | Sonora | no | soil | 10 | 12.4 | 1.19 |
| Punta Cirio | Sonora | no | soil | 10 | 15.4 | 2.43 |

**Table S2.** Results of the linear mixed models of island biogeographic variables of BirdIsland (sea bird or non-seabird islands) and area. Distance from mainland and type of island (oceanic or land bridge) were not significant.

|  |  | **Estimate** | **Std. Error** | **df** | **t value** | **Pr(>\|t\|)** |
| --- | --- | --- | --- | --- | --- | --- |
| Soil |  |  |  |  |  |  |
|  | intercept | 24.61 | 2.52 | 13.96 | 9.76 | <0.0001 |
|  | Birdisland | 9.54 | 3.00 | 13.93 | 3.18 | 0.01 |
|  | Area | 2.45 | 0.66 | 13.92 | -3.73 | 0.00 |
| Cardón |  |  |  |  |  |  |
|  | intercept | 20.72 | 2.82 | 13.97 | 7.34 | <0.0001 |
|  | BirdIsland | 9.35 | 3.35 | 13.95 | 2.79 | 0.02 |
|  | Area | -2.25 | 0.74 | 13.94 | -3.06 | 0.01 |

**Table S3.** Island biogeography variables and data used in linear mixed models for the Gulf of California islands in this study.

| **Name** | **bird island?** | **Distance from mainland (km)** | **Island type** | **Area (km^2^)** |
| --- | --- | --- | --- | --- |
| Isla Alcatraz | yes | 2.01 | Land bridge | 1.44 |
| Isla Cardonosa | yes | 19.29 | Oceanic | 0.14 |
| Isla Carmen | no | 6.03 | Land bridge | 143.03 |
| Isla Catalina | no | 25.16 | Oceanic | 39.7 |
| Isla Cerralvo | no | 8.73 | Oceanic | 140.46 |
| Isla Cholludo | yes | 1.09 | Land bridge | 0.2 |
| Isla Dátil | no | 1.94 | Land bridge | 1.25 |
| Isla Las Ánimas | yes | 16.36 | Oceanic | 4.26 |
| Isla Partida | yes | 17.88 | Oceanic | 1.36 |
| Isla Rasa | yes | 20.79 | Oceanic | 0.68 |
| Isla Salsipuedes | yes | 19.21 | Oceanic | 1.16 |
| Isla San Diego | no | 19.06 | Land bridge | 0.6 |
| Isla San Esteban | no | 11.64 | Oceanic | 41 |
| Isla San Jose | no | 4.16 | Land bridge | 187.6 |
| Isla San Lorenzo | no | 16.36 | Oceanic | 33 |
| Isla San Pedro Mártir | Yes | 50 | Oceanic | 2.67 |
| Isla Tortuga | no | 36.3 | Oceanic | 11.36 |

**Table S4.** Sample locations.

| **Location** | **Latitude** | **Longitude** |
| --- | --- | --- |
| Baja California peninsula | | |
| San Felipe | 30.79621 | -115.19053 |
| Bahía San Luis Gonzaga | 29.61532 | -114.40913 |
| Valle de los Cirios | 29.23856 | -114.26710 |
| Vizcaino | 27.73338 | -113.48771 |
| Volcán Tres Virgenes | 27.49452 | -112.54288 |
| Sierra de Santa Clara | 27.12654 | -113.59658 |
| Bahía Concepción | 26.62054 | -111.70827 |
| San Javier | 25.93761 | -111.58172 |
| Magdalena Plain | 24.68436 | -111.85744 |
| Isthmus of La Paz | 24.08606 | -110.62862 |
| La Ventana | 24.03559 | -109.97953 |
| Gulf of California |  |  |
| Isla Partida (bird island) | 28.88924 | -113.04042 |
| Isla Cardonosa (bird island) | 28.88682 | -113.02845 |
| Isla Rasa (bird island) | 28.82422 | -112.98061 |
| Isla Alcatraz (bird island) | 28.81266 | -111.96928 |
| Isla Cholludo (bird island) | 28.73832 | -112.30511 |
| Isla Dátil | 28.72823 | -112.29313 |
| Isla Salsipuedes (bird island) | 28.72636 | -112.95412 |
| Isla Las Ánimas (bird island) | 28.69791 | -112.93044 |
| Isla San Esteban | 28.68763 | -112.55154 |
| Isla San Lorenzo | 28.58771 | -112.78822 |
| Isla San Pedro Mártir (bird island) | 28.37866 | -112.30672 |
| Isla Tortuga | 27.4423 | -111.89066 |
| Isla Carmen | 25.97648 | -111.13184 |
| Isla Catalina | 25.6055 | -110.77934 |
| Isla San Diego | 25.19646 | -110.7019 |
| Isla San Jose | 25.03239 | -110.70638 |
| Isla Cerralvo | 24.16103 | -109.87072 |
| Sonora |  |  |
| Punta Cirio | 29.84902 | -112.64674 |
| Kino | 28.83145 | -111.80492 |
| El Cardonal | 28.46505 | -111.67288 |
